# Patterns of change and factors associated with IADL function decline in community-dwelling older adults with arthritis

**DOI:** 10.1038/s41598-022-19791-4

**Published:** 2022-10-07

**Authors:** Jinhee Shin, Gwang Suk Kim

**Affiliations:** 1 College of Nursing, Woosuk University, Wanju, Jeollabuk-do 55338 Republic of Korea; 2 Mo-Im Kim Nursing Research Institute, College of Nursing, Yonsei University, 50-1 Yonsei-ro Seodaemun-gu, Seoul, 03722 Republic of Korea

**Keywords:** Health care, Risk factors

## Abstract

Arthritis is a major cause of functional decline, which affects the quality of life (QoL) of older adults. This study analyzed instrumental activities of daily living (IADL) patterns in older adults with arthritis and the risk factors of functional decline. Data from the Korean Longitudinal Study of Aging (KLoSA), in which the participants were community-dwelling older adults aged ≥ 65 years and conducted every two years, were used to examine patterns in IADL performance between 2006 and 2016. The participants comprised 1,822 older adults, divided into an arthritis group and a non-arthritis group. A Generalized Estimating Equations (GEE) model and Kaplan–Meier analysis was used for the data analysis. The arthritis groups showed a statistically significant decrease in IADL function in 2012 (β = 1.283, *p* = 0.026), 2014 (β = 1.323, *p* = 0.028), and 2016 (β = 1.484, *p* = 0.014). The GEE model identified psychological conditions (depressive symptoms, cognitive function) and number of chronic diseases in the arthritis group as risk factors for increased IADL dependence. Healthcare providers should develop strategies to manage long-term functional decline, including programs to manage and prevent chronic diseases, cognitive function decline, and keep depressive symptoms under control, beginning within six years of arthritis diagnosis.

## Introduction

Owing to the drastic increase in human life expectancy worldwide, there has been a growing focus on medical services to prevent functional decline and diseases among older adults and improve their quality of life (QoL)^1–3^. Arthritis is a chronic disease that develops with age and is common among older adults. It causes joint inflammation and pain, limits joint range of motion, and affects the musculoskeletal system^4^. The worldwide prevalence of arthritis is increasing. The prevalence of rheumatoid arthritis is 0.5–1% worldwide and 1.1–2.1% in South Korea^5^. Over 14 million people, including one million racial and ethnic minorities in the United States, have osteoarthritis, with more than half of them being ≥ 65 years of age^6^. The prevalence of degenerative osteoarthritis is reported at 9.3% in Korean women and 28.5% in Korean men^7^. Arthritis includes osteoarthritis and rheumatoid arthritis types, with osteoarthritis being the most common^8^.

Arthritis and other rheumatoid arthritis (hereinafter called arthritis) are a major cause of functional disabilities^9^. A study of the Austrian population identified arthritis as the major cause of functional decline^10^, and many studies have identified age as a major risk factor that accelerates disability in instrumental activities of daily living (IADL)^11^. About 60% of older adults aged ≥ 65 years develop arthritis, and one in ten experience difficulties conducting daily activities^12^. Although IADL function declines over time in older adults, those with arthritis experience more severe IADL impairment. If not properly treated, arthritis can limit daily activities and cause physical problems such as joint deformities^13^. Progressive functional decline leads to functional disabilities, increases in healthcare costs for older adults, and affects their QoL. Therefore, functional decline in older adults is an important public health concern that must be prevented.

Functioning is a comprehensive concept that includes activities, such as self-care, productivity, leisure, and rest^10^. IADLs are activities that allow individuals to live independently in a community, including grooming, housekeeping, preparing food, doing laundry, using transportation, handling money, using a phone, shopping, traveling short distances, and taking medications^14^. They involve various types of interactions, such as social activities in different environments, and require more complex cognitive and motor skills than basic ADLs. Therefore, it is more suitable to assess the IADL performance of community-dwelling individuals with arthritis by measuring their ability to adapt or maintain independent living, rather than assessing their basic ADL performance^14^.

The predictors of functional decline based on IADLs in older adults living in a community are age, low education levels, previous history of hospitalization, and cognitive decline^15^. The risk factors for functional decline, in addition to age, are chronic disease, dysfunction in the upper and lower limbs, body mass index (BMI), lack of physical activity, smoking, alcohol use, and low levels of social activity^16^. IADL functional decline is strongly associated with cognitive disability in community-dwelling older adults^17^. As depression increases, so does the risk of IADL functional decline^18^. Therefore, it is necessary to check the functional decline of older adults with arthritis and identify risk factors that allow more appropriate prevention and management programs^19^.

Studies conducted on IADL patterns and their predictors among community-dwelling older adults^20^ reported an association between health (mental/functional limitations/number of health conditions) and IADL performance^21^. The association between IADL performance and stress in older adults with arthritis has also been examined^22^. A few studies focused on IADL patterns and risk factors among older adults with arthritis over the long term at the national level. This longitudinal study aimed to examine patterns related to IADL performance and risk factors of functional decline in community-dwelling older adults with arthritis for over 10 years. It provides insights to develop effective health promotion strategies and public health care programs by identifying the factors that this population can use to maintain their functional abilities, thereby preventing functional decline.

## Methods

### Data source and participants

This study employed a secondary analysis using Korean Longitudinal Study of Aging (KLoSA) data. The KLoSA was initiated by the Korea Labor Institute in 2006 to obtain basic data on South Korea’s aging population. The KLoSA is a longitudinal study that has followed a sample every two years since 2006 using the same questionnaire. Individuals who met the study’s criteria were added to the sample, and new data were collected due to the aging of the study’s sample. The participants of the KLoSA are Koreans aged ≥ 45 years. They were randomly selected using a multi-stage, stratified probability sampling design to obtain a sample representative of the Korean population. The questionnaire used investigated factors related to the population, family, health status, employment, income, expenses, assets, and subjective expectations. A trained researcher conducted computer-assisted personal interviews to collect the data. The first (2006) to sixth (2016) datasets were used in this study. The KLoSA database is open to the public and can be downloaded anonymously from the Korea Labor Institute website. The KLoSA was performed after obtaining informed consent from the participants. This study ensured anonymity, confidentiality, and that there was no potential harm to the participants. It was conducted with the approval of the Institutional Review Board (Y-2020-0226).

**Figure 1:**
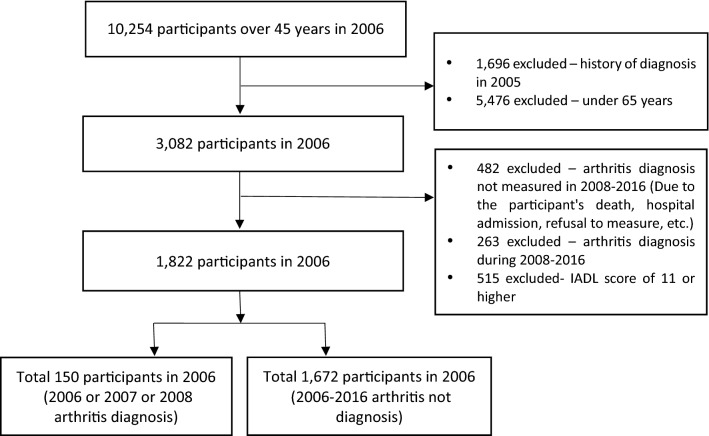
Participant selection process in the Korean Longitudinal Study of Aging.

The first survey (2006) comprised 10,254 participants. Of these, 1,696 participants diagnosed with arthritis before 2005 and 5,476 participants aged ≤ 65 years were excluded, resulting in 3,082 participants eligible for inclusion. Of these, 482 participants were excluded for reasons such as the participant’s death, hospital admission, or refusal to measure. Furthermore, participants with newly diagnosed arthritis between 2008 and 2016 (*n* = 263) were excluded. Therefore, we intended to reduce the risk of classification bias (e.g., those diagnosed with arthritis and the non-diagnosed) due to diagnostic inaccuracies for the six wave periods^23^. Additionally, participants without physical limitations (IADL score of 10) were selected, and those scoring 11 or higher were excluded (*n* = 515). Finally, 150 newly diagnosed people with arthritis between the 2006 and 2008 surveys and 1,672 participants who were not diagnosed with arthritis between the 2006 and 2016 surveys were included (Fig. 1).

### Variables

#### Arthritis diagnosis

An arthritis diagnosis refers to a case diagnosed by a doctor based on the criteria for an arthritis diagnosis (such as the patient's symptoms, physical examination, imaging tests (X-ray or MRI), and joint fluid tests). Participants who responded with “yes” or “no” to “Have you been diagnosed with arthritis or rheumatoid arthritis by a physician?” in the 2006–2016 survey were included. Participants newly diagnosed with arthritis were those who answered that they had been newly diagnosed in the 2008 survey.

#### Instrumental activities of daily living (IADL)

The Korean Instrumental Activities of Daily Living (K-IADL) scale was used to measure IADL in the KLoSA. It consists of 10 items: grooming, housekeeping, preparing food, doing laundry, using transportation, handling money, phone use, shopping, traveling short distances, and taking medications. The responses to these items were on a 3-point scale: “1 = *completely independent*,” “3 = *partially dependent*,” and “5 = *completely dependent*.” Total scores ranged between 10 and 50, with higher scores indicating higher dependence^24^. In this study, the survey time point when the IADL score was 11 or higher (partially dependent and completely dependent), indicating the point at which the first decline at each survey occurred, was noted as the time point of the onset of IADL decline events.

#### Cognitive function

The Korean version of the Mini-Mental State Examination (K-MMSE) was used to measure cognitive functioning in the KLoSA. The K-MMSE assesses orientation, memory, concentration, calculation, recall, language, and visuospatial construction. The final score ranges between 0 and 30 points. Higher total scores indicated higher levels of cognitive functioning. Normal cognitive functioning is indicated by a score of ≥ 24^25,26^.

#### Depressive symptoms

In the KLoSA, depression was measured using the Korean version of the Center for Epidemiologic Studies Depression Scale (CES-D10). The CES-D10 contains 10 items: three on depression, five on somatic symptoms, and two on positive affect. The two items of positive emotion were calculated by reversing the score inversely to the inverse items (to re-code the responses so that a high score is transformed into the corresponding low score on the scale). The final score ranged between 0 and 10 points. Higher total scores indicated higher levels of depression^27^.

#### Demographic characteristics

Age was considered a continuous variable, and gender was categorized into male and female. Education levels were categorized into elementary school, middle school, high school, and college graduation or above. Marital status was categorized into “yes (married)” and “no (divorced, widowed, or single).” Residential areas were categorized into large city, medium city, and rural areas.

#### Health status

BMI was calculated by dividing the body weight (kg) by the squared height (m^2^) and was analyzed as a continuous variable. The number of chronic diseases was calculated by adding one for each of these nine diseases: hypertension, diabetes, cancer, chronic lung disease, liver disease, heart disease, cerebrovascular disease, mental disorder, and prostate disease. The total count ranged from zero to nine.

#### Health behaviors

The participants responded “yes” if they exercised regularly at least once per week and “no” if not. They were categorized into “non-smokers” and “current smokers” and into “non-drinkers” and “current drinkers,” depending on their alcohol use and smoking habits, respectively.

### Data analyses

Data were analyzed using Stata 16.0/SE version (Stata Corp., College Station, Texas). The demographic characteristics, health status, and health behaviors of the participants since 2006 were expressed in percentages and means. The generalized estimation equations (GEE) model, a general statistical method for fitting marginal models to longitudinal data in biomedical research, was used to analyze changes in IADL over time from 2006 to 2016, for both groups. In the correlation structure assumptions of the GEE model, the regression coefficients were set as “exchangeable^28,29^. The GEE model output showed the unstandardized regression coefficients. GEE is a general statistical method for fitting marginal models to longitudinal data in biomedical research. It considers the correlations between repeated measures for the same entities in longitudinal data and is also used for data with missing values^30,31^. GEE is a population-level model based on the quasi-likelihood function approach that can employ random effects to capture correlations between multiple observations on the same participants^32^. Patterns in participants’ IADL changes over time were examined. The time course of change in IADL scores was analyzed using the Kaplan–Meier (K–M) method. The significance of the curves was tested using the log-rank test. Furthermore, GEE was used to analyze the risk factors for the IADL score. *p*-value < 0.05 was considered statistically significant.

### Ethics approval and consent to participate

The study was conducted with the approval of the National Statistical Office (Approval no.: 33,602). The KLoSA was conducted after acquiring informed consent from the participants. The KLoSA database has been released to the public for scientific use. Furthermore, this study ensures anonymity, confidentiality, and that there is no potential harm to the participants. Moreover, approval of the Institutional Review Board of Yonsei University Health System was obtained prior to the secondary data analysis (Y-2020–0226).

## Results

### General characteristics

A total of 1822 older adults with normal IADL scores were included in the study between 2006 and 2008. Of these older adults, 150 were newly diagnosed with arthritis, whereas 1672 were not diagnosed with arthritis. Table 1 shows the group differences in demographic characteristics in 2006. The participants’ mean age was 71.69 (SD = 5.46) years; the mean age for participants with arthritis and non-arthritis was 71.21 and 71.73 years, respectively. Most participants completed only elementary school (66.1%) and were married (70.9%). Moreover, most participants were non-smokers (67.5%) and non-drinkers (80.7%). The overall mean CES-D10 and K-MMSE scores were 1.42 (SD = 1.72) and 24.24 (SD = 5.12), respectively. For participants with arthritis and non-arthritis, the mean CES-D10 was 1.77 (SD = 2.14) and 1.39 (SD = 1.67), and the K-MMSE was 23.26 (SD = 5.53) and 24.32 (SD = 5.08), respectively.

**Table 1:** Comparison of characteristics between the arthritis and non-arthritis groups in 2006.

Variables	Categories	Total (*n* = 1822) mean ± SD or *n*(%)	Arthritis^a^ (*n* = 150) mean ± SD or *n*(%)	Non-arthritis^b^ (*n* = 1672) mean ± SD or *n*(%)	Differences *t* or *χ*^2^	*p*
Age		71.69 ± 5.46	71.21 ± 4.91	71.73 ± 5.50	− 1.127	0.95
Gender	Male	966 (53.0)	31 (20.7)	935 (55.9)	68.683	< .001
	Female	856 (47.0)	119 (79.3)	737 (44.1)		
Education level	Elementary school	1205 (66.1)	120 (80.0)	1085 (64.9)	16.198	0.001
	Middle school	205 (11.3)	14 (9.3)	191 (11.4)		
	High school	279 (15.3)	13 (8.7)	266 (15.9)		
	College or over	132 (7.2)	3 (2.0)	129 (7.7)		
Marital status	Yes	1291 (70.9)	93 (62.0)	1198 (71.7)	6.208	0.013
	No	531 (29.1)	57 (38.0)	474 (28.3)		
Present job	Yes	427 (23.4)	25 (16.7)	402 (24.0)	4.174	0.041
	No	1395 (76.6)	125 (83.3)	1270 (76.0)		
Residential area	Large city	728 (40.0)	56 (37.3)	672 (36.9)	1.255	0.534
	Medium city	535 (29.4)	50 (33.3)	485 (29.0)		
	Rural areas	559 (30.7)	44 (29.3)	515 (30.8)		
Smoking	Non-smokers	1230 (67.5)	137 (91.3)	1333 (79.7)	29.558	< .001
	Current smokers	592 (32.5)	13 (8.7)	339 (20.3)		
Drinking	Non-drinkers	1470 (80.7)	119 (79.3)	1091 (65.3)	22.785	< .001
	Current drinker	325 (19.3)	31 (20.7)	581 (34.7)		
Regular exercise	Yes	678 (37.2)	59 (39.3)	619 (37.0)	0.315	0.575
	No	1144 (32.8)	91 (60.7)	1053 (63.0)		
Depressive symptoms (CES-D10)		1.42 ± 1.72	1.77 ± 2.14	1.39 ± 1.67	2.597	0.010
Cognitive function (K-MMSE)		24.24 ± 5.12	23.26 ± 5.53	24.32 ± 5.08	− 2.413	0.016
BMI		22.69 ± 2.77	23.25 ± 2.49	22.64 ± 2.79	2.521	0.012
Number of chronic diseases		0.73 ± 0.85	0.75 ± 0.72	0.73 ± 0.86	0.310	0.756

^a^2006–2008 arthritis diagnosis.

^b^2006–2016 arthritis not diagnosis.

*BMI* body mass index, *CES-D10* Center for Epidemiologic Studies Depression Scale, *MMSE* Mini Mental State Examination, *SD* Standard deviation.

### Changes in IADL item scores over time

Table 2 presents the results of the IADL scores according to the group and over time. In terms of IADL changes over time, IADL scores in 2010 (β = 0.577, *p* = 0.001), 2012 (β = 0.542, *p* = 0.004), 2014 (β = 0.623, *p* = 0.002), and 2016 (β = 1.088, *p* < 0.001) were statistically significantly greater than those in 2006, confirming the influence of time. Furthermore, the higher IADL score in 2012 (β = 1.283, *p* = 0.026), 2014 (β = 1.323, *p* = 0.028), and 2016 (β = 1.484, *p* = 0.014) indicated that the arthritis group had a statistically significantly greater interaction between groups and time.

**Table 2:** Generalized estimating equations analyses of IADL function decline (*n* = 1822).

	Adjusted			
	Coefficient	*SE*	95% CI	*p*-value
**Change of IADL**				
Arthritis group (ref. non-arthritis)	0.013	0.424	− 0.818 to 0.845	0.975
Time (year) 2008 (ref. Time 2006)	0.179	0.163	− 0.141 to 0.499	0.273
Time 2010 (ref. Time 2006)	**0.577**	**0.176**	**0.230 to 0.924**	**0.001**
Time 2012 (ref. Time 2006)	**0.542**	**0.190**	**0.168 to 0.916**	**0.004**
Time 2014 (ref. Time 2006)	**0.623**	**0.205**	**0.221 to 1.025**	**0.002**
Time 2016 (ref. Time 2006)	**1.088**	**0.224**	**0.648 to 1.527**	** < 0.001**
**Arthritis group ∗ time (ref. non-arthritis group ∗ time 2006)**				
Arthritis group ∗ time 2008	− 0.419	0.542	− 1.482 to 0.643	0.440
Arthritis group ∗ time 2010	0.732	0.557	− 0.359 to 1.824	0.189
Arthritis group ∗ time 2012	**1.283**	**0.575**	**0.156 to 2.410**	**0.026**
Arthritis group ∗ time 2014	**1.323**	**0.601**	**0.145 to 2.501**	**0.028**
Arthritis group ∗ time 2016	**1.484**	**0.605**	**0.297 to 2.671**	**0.014**

Adjusted covariates: age, gender, education level, marital status, present job, residential area, BMI, number of chronic diseases, MMSE, CES-D10, regular exercise, smoking, and drinking in 2006.

*IADL* instrumental activities of daily living, *SE* standard error, *CI* confidential interval, *SE* standard error.

Significant values are in bold.

**Figure 2:**
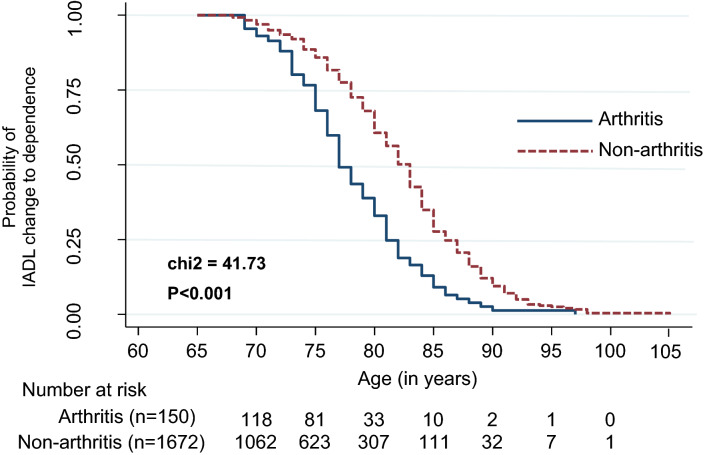
Kaplan–Meier (K–M) curve (log-rank test) illustrating the effects of arthritis over time on IADL decline (*n* = 1822).

Figure 2 shows the K–M cumulative probability curve of the probable incidence of IADL decline or the time to change probability of functional independence in IADL. The IADL independence was statistically significantly lower in the arthritis group (χ^2^ = 41.73, *p* < 0.001). The K–M cumulative probability curve of the probable incidence of IADL score on each item level was statistically significantly different (Supplementary Fig. S1 online). The IADL independence was significantly lower in the arthritis group, from independence in IADL to dependence for these seven activities: shopping (χ^2^ = 9.08, *p* = 0.003); housekeeping (χ^2^ = 6.32, *p* = 0.012); food preparation (χ^2^ = 6.65, *p* = 0.009); laundry (χ^2^ = 5.54, *p* = 0.019); going out (χ^2^ = 6.90, *p* = 0.009); mode of transportation (χ2 = 15.91, *p* = 0.001); and phone use (χ^2^ = 4.35, *p* = 0.037).

### Risk factors for IADL function decline

A Cox regression model was used to identify the risk factors affecting IADL functional decline (Table 3). For the non-arthritis group, a protective factor was regular exercise (yes as reference group, Hazard Ratio (HR) = 1.253, 95% CI 1.153–1.362, *p* < 0.001). The risk factors were age (HR = 1.028, 95% CI 1.020–1.036, *p* < 0.001), gender (male [HR = 0.560, 95% CI 0.574–0.736, *p* < 0.001]), job (yes as reference group, HR = 1.153, 95% CI 1.039–1.279, *p* = 0.007), living in a residential area (large city as the reference group, rural areas: HR = 1.234, 95% CI 1.100–1.385, *p* < 0.001), drinking (current drinkers as reference group, HR = 0.761, 95% CI 0.689–0.841, *p* < 0.001), number of chronic diseases (HR = 1.155, 95% CI 1.105–1.208, *p* < 0.001), depressive symptoms (CES-D10; HR = 1.070, 95% CI 1.056–1.084, *p* < 0.001), cognitive function (K-MMSE; HR = 0.894, 95% CI 0.887–0.901, *p* < 0.001), and BMI (HR = 0.974, 95% CI 0.959–0.989, *p* < 0.001).

**Table 3:** Risk factors of IADL functional decline of non-arthritis vs. arthritis groups.

Variables	Non-arthritis (n = 1672)			Arthritis (n = 150)		
	Exp (B)	95% CI	*p*	Exp (B)	95% CI	*p*
Age	**1.028**	**1.020–1.036**	** < 0.001**	**1.050**	**1.026–1.091**	** < 0.001**
Gender (ref: female)	**0.560**	**0.574–0.736**	** < 0.001**	0.977	0.571**–**1.673	0.934
**Education level (ref: College or higher)**						
Elementary school	1.039	0.850**–**1.271	0.706	0.559	0.170**–**1.839	0.395
Middle school	0.983	0.794**–**1.218	0.881	0.637	0.202**–**2.003	0.441
High school or higher	0.935	0.633**–**1.381	0.736	0.475	0.164**–**1.377	0.171
Marital status (ref: No)	1.068	0.800**–**1.195	0.828	1.167	0.817**–**1.667	0.395
Present job (ref: Yes)	**1.153**	**1.039–1.279**	**0.007**	1.376	0.893**–**2.119	0.147
**Residential area (ref. Large city)**						
Medium city	1.025	0.908**–**1.158	0.684	0.924	0.632**–**1.352	0.687
Rural areas	**1.234**	**1.100–1.385**	** < 0.001**	0.884	0.586**–**1.333	0.559
**Smoking (ref. non-smokers)**						
Current smokers	0.905	0.689**–**0.841	0.111	0.688	0.376**–**1.259	0.225
**Drinking (ref. current drinker)**						
Non-drinkers	**0.761**	**0.689–0.841**	** < 0.001**	1.134	0.710**–**1.812	0.596
**Regular exercise (ref. Yes)**						
No	**1.253**	**1.153–1.362**	** < 0.001**	1.143	0.838**–**1.561	0.786
Number of chronic diseases	**1.155**	**1.105–1.208**	** < 0.001**	**1.311**	**1.142–1.504**	** < 0.001**
Depressive symptoms (CES-D10)	**1.070**	**1.056–1.084**	** < 0.001**	**1.070**	**1.020–1.222**	** < 0.001**
Cognitive function (K-MMSE)	**0.894**	**0.887–0.901**	** < 0.001**	**0.901**	**0.877–0.926**	** < 0.001**
BMI	**0.974**	**0.959–0.989**	**0.001**	1.005	0.946**–**1.067	0.862
	Prob > chi^2^ = < 0.001			Prob > chi^2^ = < 0.001		

Significant values are in bold.

*IADL* instrumental activities of daily living, *BMI* body mass index, *CES-D10* Center for Epidemiologic Studies Depression Scale, *MMSE* Mini Mental State Examination, *CI* confidential interval.

The risk factors for the arthritis group were age (HR = 1.050, 95% CI 1.026–1.091, *p* < 0.001); number of chronic diseases (HR = 1.311, 95% CI 1.142–1.504, *p* < 0.001); depressive symptoms (CES-D10; HR = 1.070, 95% CI 1.020–1.222, *p* < 0.001), and cognitive function (K-MMSE; HR = 0.901, 95% CI 0.877–0.926, *p* < 0.001).

## Discussion

Maintaining the ability to perform ADLs independently is crucial for older adults with chronic diseases^11^. IADL functioning in older adults is of substantial interest given its prominent role in independent living in “successful” aging. In this study, IADL functioning decreased over time, with different perspective variations. IADL scores significantly differed over time among the arthritis and non-arthritis groups. In the arthritis group, the IADL functioning steadily decreased after six years of diagnosis, increasing IADL scores over time.

Given that the survey is conducted every two years, each participant’s point of functioning decline varies, as some begin at 6 years while others begin at 10 years. In an Australian longitudinal study using three-year cohort data, older women with musculoskeletal/somatic diseases had a slow increase in IADL score over time, the highest being between six and nine years^33^. Based on prior research and the results of this study, it is suggested that interventions to prevent and manage IADL functioning should be started early, at least within six years after diagnosis. Active early intervention will reduce the occurrence of functional decline among community-dwelling older adults^34^.

This study found that risk factors for increased IADL score in the non-arthritic group were related to age, gender, present job, residential area, drinking, exercise, number of chronic diseases, depressive symptoms, cognitive function, and BMI. As age increases, the likelihood of recovering from functional decline decreases^35^. In this study, age was identified as a risk factor for disability in IADL, and IADL scores indicated a higher dependence over time. Furthermore, a prospective cohort study on IADL disability among older adults found that the risk of functional decline drastically increased with age^11^, which is consistent with the present results. Previous studies have defined disability as an inability to perform ADL or IADL, and found that older adults with chronic conditions and women are at a higher risk of developing a disability^36,37^. Older adults living in rural areas have limited access to knowledge regarding the prevention and timely treatment of chronic diseases; a lack of economic resources; and earlier impairments in physical function due to strenuous farm work^38^. It is a well-known that chronic diseases are strongly associated with functional decline. In the systematic review of risk factors for impaired functional status among community-dwelling older people, there is evidence that excessive drinking compared to moderate drinking is associated with an increased risk of functional decline^16^.

In this study, higher BMI was associated with a lower IADL score. A study on potential profile analysis of daily life activities of Chinese older adults found that those who were underweight or obese may have a higher risk of combined basic activity of daily living (BADL) and IADL impairment^39^. In a systematic study on risk factors for the functional status decline of community-dwelling older adults, the strongest evidence for an increased risk of function decline was increased and decreased BMI^16^. The positive association between obesity and high ADL impairment may be explained by an increased likelihood of chronic disease in obese people, further limiting their physical function^40^. On the contrary, low body weight is associated with bone mineral density and ADL damage due to decreased bone mineral density^41^. Therefore, future studies should identify the relationship between BMI level (under, normal, and overweight) and ADL/IADL function in Korean older adults. A longitudinal trajectory study examining the IADL performance of middle-aged older adults in Taiwan reported that the participants engaging in sufficient levels of exercise were more likely to experience late-onset disabilities^18^. In addition, physical activities improve joint health and function while also reducing the risk of joint-related disabilities^42^. Therefore, risk factors such as being a woman, living in a rural area, having a chronic disease, drinking, and minimal physical activity are not new discoveries. However, the factor of “present job” is controversial. In the case of present job, in a cross-sectional and longitudinal analysis of job losses of middle-aged and older adult Koreans, health problems related to physical disabilities had the greatest impact on leaving the workplace^41^. Furthermore, IADL disability affected job loss and retirement because the onset of a disability may be the deciding factor for leaving the workplace, as most older workers in Korea have been engaged in physical labor (66.2%)^43^. Therefore, this study identified not working as a risk factor. However, it may indicate that the job was lost due to loss of function.

This study’s intriguing finding was that the risk factors associated with IADL change in the arthritis and non-arthritis groups were not equal. Number of chronic diseases, cognitive decline, and depressive symptoms were risk factors for IADL scores increasing in the arthritis group, which are the same risk factors as those in the non-arthritis group. The number of chronic diseases is strongly associated with decreased physical function. One study reported a significant interaction between gait disability and the number of chronic diseases^11^. Chronic diseases may not be detected until complications arise, resulting in additional negative health consequences^44^. Furthermore, as chronic diseases are co-occurring multimorbidities, monitoring older adults’ overall health conditions and functioning is necessary for them to achieve a longer life expectancy and healthy aging^45^. In this study, cognitive function scores were associated with IADL scores. Some issues in cognitive function and IADL require further discussion. Whether cognitive impairment is a cause of physical functional decline or simply increases its likelihood has not been determined. For example, cognition was found to be a predictor of physical function in some studies^46–48^, whereas physical function was a predictor of cognition decline in others^49,50^. The question of interest is whether cognitive decline leads to subsequent physical decline, whether physical decline leads to subsequent cognitive decline, or whether the decline in function mutually influences each other. Studies investigating the association between arthritis and cognitive functioning reported that arthritis increased the risk of cognitive deficits^51^. In retrospective cohort studies, patients with osteoarthritis were 25% more likely to have dementia than those without osteoarthritis^51^. Furthermore, those with rheumatoid arthritis were twice as likely to have cognitive deficits as those without rheumatoid arthritis^52^. These pathways are driven by various underlying biological hypotheses that explain how cognition and physical functions are interrelated. In this hypothesis, the association may be caused by central nervous system changes that initially appear as a physical functional decline and later as a cognitive decline^52,53^. Moreover, chronic inflammation, mental disease, or underlying pathology can cause simultaneous cognitive and physical functional decline^54^.

However, despite these controversies, this study identified cognitive impairment as a risk factor for impaired physical function. This is because not all participants in our study had IADL impairment at baseline and cognitive impairment occurred before the onset of physical decline. Therefore, it is important to carefully monitor and prevent cognitive decline in older adults with arthritis. Although the association mechanism between arthritis and depression is not fully understood, selected studies provide useful evidence. First, there is a pathophysiological mechanism of association between depression and arthritis. Some evidence suggests that pro-inflammatory biomarkers such as cytokines and C-reactive protein are associated with depression^55^. This link between inflammation and depression increases the risk of depression in individuals with inflammatory conditions such as rheumatoid arthritis^56^. Functional limitations in people with arthritis may impair behavioral changes associated with reduced depression, such as physical activity and occupational and social activities^57^. Depressive symptoms may be present in 13–20% of arthritis patients^58^. However, depression in arthritis patients remains unrecognized and untreated, mainly in clinical practice^59^. Furthermore, depression has a strong positive association with the risk of cognitive decline^60^, although it is not yet clear whether it is a cause of cognitive decline or increases its likelihood. However, chronic long-term depression impairs the brain and results in decreased function^61^. According to the present findings, the strong factors influencing the lowering of IADL in the arthritis group are the number of chronic diseases, depressive symptoms, and cognitive decline. These factors are strongly associated. Therefore, it is essential for older adults diagnosed with arthritis to follow up, prevent, and treat cognitive decline and depression symptoms to prevent IADL decline. In the future, when implementing a program for older adults with arthritis, this study proposes the application of content and strategies to control chronic diseases, depressive symptoms, and cognitive decline after screening individual risk factors related to IADL.

This study has some limitations. First, in this study, we used information from the participants who responded that they had been diagnosed with arthritis by a doctor. Therefore, an accurate diagnosis of arthritis or exclusion of false diagnosis cannot be fully guaranteed due to the lack of diagnostic criteria and information for arthritis (exact category or severity). Second, this study could not determine the cause of arthritis due to the limitations of the data. Variables that can affect the occurrence of arthritis or changes in the state of arthritis were adjusted to minimize these limitations. Third, there is the possibility of selection bias due to differences in participants’ inclusion and exclusion characteristics. Furthermore, there may be potential recall bias and consequences of misclassification bias due to memory distortion and false reporting on the part of older adults. Finally, this study analyzed changes over time in IADL scores using the Kaplan–Meier (K-M) method. The IADL change was analyzed as the time point of the onset of IADL decline events. However, IADL change may not be a sufficient criterion to be clinically meaningful.

## Conclusion

IADL patterns over 10 years among Korean older adults aged ≥ 65 years who were diagnosed with arthritis were examined. Furthermore, the risk factors affecting their IADL function decline were identified and the patterns of changes in each IADL item were examined. IADL function was significantly decreased in the arthritis group compared to the non-arthritis group. The significant difference between the two groups was generally 6 years after the arthritis diagnosis. Risk factors affecting IADL function decline in the arthritis group were depressive symptoms and cognitive decline. These study results can be used to develop a personalized program for the long-term management of these risk factors in older adults with arthritis. Consequently, it is necessary to participate in a management program for IADL, manage chronic diseases and depressive symptoms, receive regular examinations, and apply preventive measures for cognitive functional decline before six years have passed since the diagnosis of arthritis.

## Supplementary Information

Supplementary Figure 1.

## Data Availability

The data from the KLoSA are publicly available for download on the KLoSA website (https://survey.keis.or.kr/klosa/klosa01.jsp).
