# Supplementary material for: Patterns of change and factors associated with IADL function decline in community-dwelling older adults with arthritis
Source: Sci Rep. 2022 Oct 7;12:16840. doi: 10.1038/s41598-022-19791-4 (PMC9546837; doi:10.1038/s41598-022-19791-4)
Supplement: Supplementary file 1 — Supplementary Figure 1. [file 41598_2022_19791_MOESM1_ESM.docx]

Patterns of Change and Factors Associated with IADL Function Decline in Community-Dwelling Older Adults with Arthritis

Jinhee Shin^1^, Gwang Suk Kim^2*^

^1^College of Nursing, Woosuk University, Wanju, Jeollabuk-do, 55338, Republic of Korea, Email: [jini2112@yonsei.ac.kr](mailto:jini2112@yonsei.ac.kr)

^2^Mo-Im Kim Nursing Research Institute, College of Nursing, Yonsei University, 50-1 Yonsei-ro Seodaemun-gu, Seoul, 03722, Republic of Korea, Email: gskim@yuhs.ac

***Correspondence:**

Gwang Suk Kim

Mo-Im Kim Nursing Research Institute, College of Nursing, Yonsei University, Seoul, South Korea

50-1 Yonsei-ro, Seodaemoon-Gu, Seoul 03722, Republic of Korea

Email: gskim@yuhs.ac

Phone: 82-2-2228-3342

Fax: 82-2-392-5440

**Supplementary Figure S1**. Kaplan-Meier (K-M) curve (log-rank test) illustrating the effects of arthritis over time for IADL item scores (*n*=1,822): **(a)** shopping, **(b)** housekeeping, **(c)** food preparation, **(d)** laundry, **(e)** going out, **(f)** mode of transportation, **(g)** grooming, **(h)** handling money, **(i)** phone use, **(j)** responsibility for own medications

| **(a) shopping** |
| --- |
| 0  .  0  0  0  .  2  5  0  .  5  0  0  .  7  5  1  .  0  0  1008  566  260  91  33  7  1  1  93  53  21  9  2  1  0  0  Number at risk  60  65  70  75  80  85  90  95  100  105  110  chi2 = 9.08  *P* = 0.003  Arthritis (n=150)  Non-arthritis (n=1672)  Age (in years)  Non-arthritis  Arthritis  Probability of shopping  change to dependence |
| **(b) housekeeping** |
| 0  .  0  0  0  .  2  5  0  .  5  0  0  .  7  5  1  .  0  0  991  543  242  71  20  5  2  1  85  48  19  6  1  0  0  0  Number at risk  60  65  70  75  80  85  90  95  100  105  110  Age (in years)  Non-arthritis  Arthritis  chi2 = 6.32  *P* = 0.012  Probability of housekeeping  change to dependence  Arthritis (n=150)  Non-arthritis (n=1672) |
| **(c)** **food preparation** |
| 0  .  0  0  0  .  2  5  0  .  5  0  0  .  7  5  1  .  0  0  1019  552  250  83  24  5  1  90  51  21  8  2  1  0  Number at risk  105  60  65  70  75  80  85  90  95  100  Age (in years)  Non-arthritis  Arthritis  chi2 = 6.65  *P* = 0.009  Probability of food preparation  change to dependence  Arthritis (n=150)  Non-arthritis (n=1672) |
| **(d) laundry** |
| 0  .  0  0  0  .  2  5  0  .  5  0  0  .  7  5  1  .  0  0  1025  569  270  95  36  5  1  1  92  55  19  9  2  1  0  0  Number at risk  60  65  70  75  80  85  90  95  100  105  110  Non-arthritis    Arthritis  chi2 = 5.54  *P* = 0.019  Age (in years)  Probability of laundry  change to dependence  Arthritis (n=150)  Non-arthritis (n=1672) |
| **(e) going out** |
| 0  .  0  0  0  .  2  5  0  .  5  0  0  .  7  5  1  .  0  0  1021  578  273  93  31  6  1  1  94  55  24  10  2  1  0  0  Number at risk  60  65  70  75  80  85  90  95  100  105  110  Non-arthritis    Arthritis  chi2 = 6.90  *P* = 0.009  Age (in years)  Probability of going out  change to dependence  Arthritis (n=150)  Non-arthritis (n=1672) |
| **(f) mode of transportation** |
| 0  .  0  0  0  .  2  5  0  .  5  0  0  .  7  5  1  .  0  0  1030  596  281  103  32  5  1  1  99  60  24  10  2  1  0  0  Number at risk  60  65  70  75  80  85  90  95  100  105  110  Age (in years)  Non-arthritis  Arthritis  chi2 = 15.91  *P* = 0.001  Probability of mode of transportation  change to dependence  Arthritis (n=150)  Non-arthritis (n=1672) |
| **(g) grooming** |
| 0  .  0  0  0  .  2  5  0  .  5  0  0  .  7  5  1  .  0  0  991  543  242  71  20  5  2  1  85  48  19  6  1  0  0  0  Number at risk  60  65  70  75  80  85  90  95  100  105  110  Non-arthritis  Arthritis  chi2 = 3.65  *P* = 0.056  Age (in years)  Probability of grooming  change to dependence  Arthritis (n=150)  Non-arthritis (n=1672) |
| **(h) handling money** |
| 0  .  0  0  0  .  2  5  0  .  5  0  0  .  7  5  1  .  0  0  1008  567  255  88  25  5  0  88  50  20  10  4  1  0  Number at risk  60  65  70  75  80  85  90  95  100  105  chi2 = 1.83  *P* = 0.176  Non-arthritis  Arthritis  Age (in years)  Probability of handling money  change to dependence  Arthritis (n=150)  Non-arthritis (n=1672) |
| **(i) phone use** |
| 0  .  0  0  0  .  2  5  0  .  5  0  0  .  7  5  1  .  0  0  1000  552  247  80  23  6  2  1  88  51  18  10  2  0  0  0  Number at risk  60  65  70  75  80  85  90  95  100  105  110  Non-arthritis  Arthritis  Age (in years)  chi2 = 4.35  *P* = 0.037  Probability of phone use  change to dependence  Arthritis (n=150)  Non-arthritis (n=1672) |
| **(j)** **responsibility for own medications** |
| 0  .  0  0  0  .  2  5  0  .  5  0  0  .  7  5  1  .  0  0  995  540  235  76  24  5  2  1  86  49  16  7  2  0  0  0  Number at risk  60  65  70  75  80  85  90  95  100  105  110  Age (in years)  chi2 = 3.30  *P* = 0.069  Non-arthritis  Arthritis  Probability of responsibility for own medications  change to dependence  Arthritis (n=150)  Non-arthritis (n=1672) |
